# Removal of Mini-Sling Anchor With Magnetic Resonance Imaging–Guided Site Marking With Methylene Blue

**DOI:** 10.1097/og9.0000000000000163

**Published:** 2026-03-19

**Authors:** Sabrina Shih, Kate Maturen, Ashley Skeith, Shane Wells, Tom Chenevert, Vikas Gulani, Daniel M. Morgan

**Affiliations:** University of Michigan Medical School, Ann Arbor, Michigan; Department of Radiology, Department of Obstetrics and Gynecology, and Department of Radiology, University of Michigan, Ann Arbor, Michigan.

## Abstract

**BACKGROUND:**

Single-incision midurethral slings are an alternative to standard midurethral slings for the treatment of stress urinary incontinence. Single-incision midurethral slings use less mesh, relying on soft tissue anchors for mesh stabilization, but can be associated with chronic pain.

**CASE:**

We describe the novel use of magnetic resonance imaging to localize and stain a retained radiolucent single-incision midurethral sling anchor with methylene blue dye. This assisted the transvaginal dissection for removal of the stained anchor for a patient with persistent chronic pelvic pain after a prior mesh removal.

**CONCLUSION:**

Marking the target anchor with dye provided crucial intraoperative guidance for the surgical dissection. This technique can be a valuable tool for localizing surgical targets that are radiolucent.

## Abstract 2

Pairing magnetic resonance imaging guidance with methylene blue dye marking is a strategy for visualization and removal of radiolucent targets during pelvic surgery.

**Teaching Points**

1. Magnetic resonance imaging (MRI) guidance can be used for presurgical site marking with methylene blue for targets that are difficult to locate radiologically or clinically.
2. Positional changes in surgery can change pelvic floor dimensions seen on imaging, highlighting the importance of presurgical marking.

Single-incision midurethral slings were introduced as a less invasive option compared with traditional slings for management of stress urinary incontinence. The data on the efficacy of single-incision midurethral slings are mixed, and they can be associated with groin pain.^1,2^ Traditional transobturator slings pass through the obturator membrane and exit through the skin of the groin. In contrast, single-incision midurethral slings are placed just inside the obturator membrane and are stabilized with polypropylene anchors that are not designed to be retracted, advanced, or removed after placement. Removal of a single-incision midurethral sling anchor from the obturator muscle–membrane complex is challenging because of its radiolucency and small size (approximately 1 cm) within dense muscle and tissue (Fig. 1B). We describe how to locate a retained Altis single-incision midurethral sling anchor with preoperative MRI-guided injection of methylene blue aiding in its surgical removal.

**Fig. 1.**
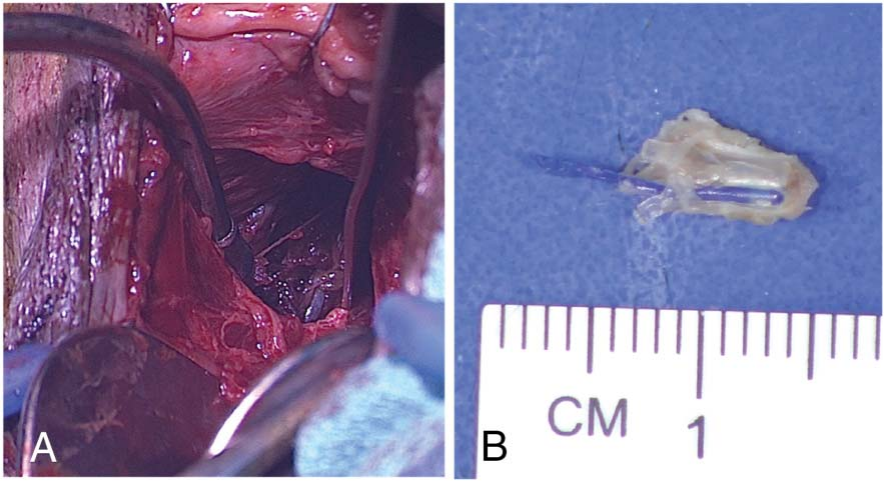
**A.** Dissection showing methylene blue dye staining of surrounding tissue. **B.** Excised midurethral sling anchor, about 1 cm in size, with attached Prolene suture.

*Shih. Radiolucent Anchor Removal With MRI Guidance. O&G Open 2026*.

## CASE

The patient gave informed consent for this case report. A 53-year-old woman presented for evaluation of chronic burning right groin and medial thigh pain and urinary urgency and frequency. Symptoms developed 1.5 years earlier, immediately after a robot-assisted total laparoscopic hysterectomy with bilateral salpingectomy, anterior and posterior repair, and Altis single-incision midurethral sling placement. One year after this initial surgery, the mesh of the sling was removed but not the anchors. Symptoms recurred within a few weeks and persisted despite pelvic floor physical therapy, mirabegron, vaginal estrogen, gabapentin, tamsulosin, and shock wave therapy. On physical examination, there were reproducible pelvic pain and urgency with palpation of the right lateral vaginal wall along the right pubic ramus. Prior pelvic computed tomography, MRI, and transvaginal ultrasound were unrevealing for the cause of this chronic pain.

**Fig. 2.**
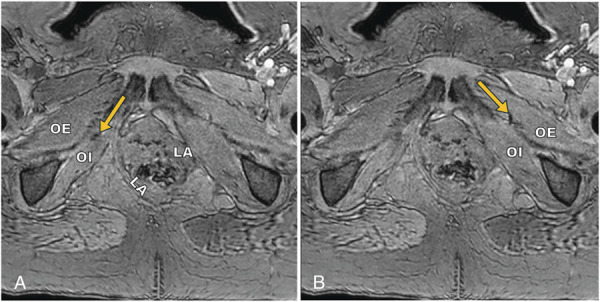
Pelvic anatomy and midurethral sling anchors visualized under T1 high-resolution isotropic volume examination, a type of three-dimensional gradient echo technique. **A.** Right anchor (*yellow arrow*) between the obturator internus (OI) and obturator externus (OE). **B.** Left anchor (*yellow arrow*) in the OE. Left levator ani (LA) is avulsed, likely from prior vaginal delivery.

Gradient echo sequences on MRI accentuate differences in the tendency of materials to become magnetized and can exaggerate the size of the signal loss from the polypropylene anchors, making it useful in initial identification of anchor location. Before preoperative imaging for this individual, we obtained an Altis sling from the manufacturer and imaged it in a water bath phantom to adjust MRI parameters to optimize anchor visibility. Pelvic MRI was then performed with a standard clinical protocol, including T2-weighted turbo spin echo with and without fat suppression, T1-weighted dual gradient echo in-phase and opposed-phase imaging, and T1-weighted gradient echo with fat suppression with and without contrast. The right anchor was identified by MRI between the obturator internus and obturator externus and the left anchor in the obturator externus (Fig. 2A and 2B). Because of the location and neuropathic character of her pain, we discussed the possibility of the retained single-incision midurethral sling anchor irritating a branch of the obturator nerve and agreed to pursue removal of the right anchor through a vaginal approach.

*Shih. Radiolucent Anchor Removal With MRI Guidance. O&G Open 2026*.

**Fig. 3.**
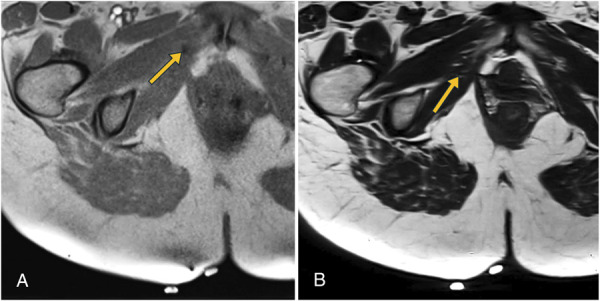
Identification of anchor on preprocedural imaging. **A.** The anchor is seen as a signal void (*yellow arrow*) on three-dimensional gradient echo (GRE) T1-weighted imaging. **B.** The anchor seen as a low-signal structure on T2-weighted turbo spin echo imaging (*yellow arrow*), corresponding to the known appearance of the anchor and the location identified on GRE.

**Fig. 4.**
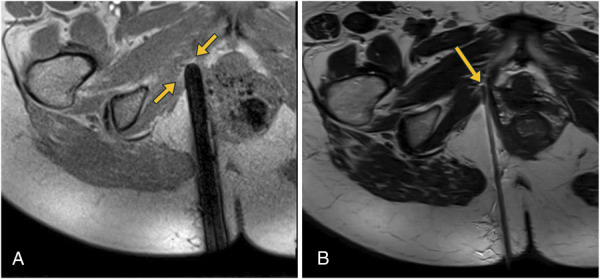
Target confirmation with the trocar adjacent to the anchor. **A.** High T1-weighted gradient echo signal attributable to enhancement from gadolinium (*yellow arrows*). **B.** High T2-weighted signal (*yellow arrow*) attributable to the water in the methylene blue dye/lidocaine/gadoteridol solution. Note the exaggerated needle artifact on T1-weighted imaging, which is why T2-weighted imaging was used for most of the needle advancement.

Given the location of the right anchor, interventional radiology was consulted to mark the right anchor with methylene blue using MRI guidance on the day of surgical removal. The patient was positioned prone on a 1.5-T scanner with vitamin E markers used to identify the site of percutaneous access. The anchor was initially identified on gradient echo (Fig. 3A), but this sequence exaggerated needle size as a result of susceptibility artifact (Fig. 4A). Therefore, an accelerated T2-weighted turbo spin echo scan was obtained with a small field of view. The anchor was identified as a signal void (Fig. 3B) that could be spatially defined in relation to the adjacent muscle by correlating to the T1-weighted imaging on which it was more apparent. A dermotomy was created, and a 16-gauge 14.4-cm trocar canula system was advanced under serial T2-weighted turbo spin echo and T1-weighted gradient echo to lie immediately medial to the anchor so that dye would be encountered before the anchor with a transvaginal surgical approach. After verifying the correct needle position, we injected about 0.1 mL of a mixture of 0.5% methylene blue, 1% lidocaine, sodium bicarbonate, and 1:150 dilution of gadoteridol gadolinium-based contrast agent. The water in the solution provided subtle increased T2-weighted signal in the tissue surrounding the anchor (Fig. 4B), and the gadolinium provided signal enhancement on gradient echo to indicate that the injected solution with methylene blue dye was present (Fig. 4A).

*Shih. Radiolucent Anchor Removal With MRI Guidance. O&G Open 2026*.

*Shih. Radiolucent Anchor Removal With MRI Guidance. O&G Open 2026*.

After MRI-guided site marking, the patient was brought to the operating room and placed under general anesthesia. With moderate Trendelenburg in lithotomy position, we made an inverted U incision in the anterior vagina. The periurethral tunnel on the right side was carried laterally until the muscle fibers of the right levator ani muscle were seen. The levator fibers were split and retracted until the obturator internus fascia was identified. Fascia and muscle fibers were split and dilated with malleable retractors until we visualized methylene blue dye (Fig. 1A). The single-incision midurethral sling anchor was palpated, grasped with a Crawford clamp, and dissected free of the tissue. The dissection was 5–6 cm lateral and anterior from the inverted U incision. We closed the split fibers of the obturator internus and levator ani with interrupted Vicryl sutures.

After the procedure, the patient reported resolution of constant groin pain. She did, however, report intermittent episodes of pelvic pain, urgency, and frequency. We hypothesized that the change from constant to intermittent pain was related to removal of the right-sided anchor and that the remaining symptoms were the result of a bladder pain syndrome. A bladder-related cause was suspected after we noted diffuse bladder mucosa bleeding during cystoscopy for bladder integrity after the anchor removal operation. She was ultimately referred to the urology department, where she had cystoscopy with fulguration of a Hunner ulcer (2 cm^2^), pelvic floor trigger point injections, and a pudendal nerve block in the 3 months after her initial operation, which improved her pain management.

## DISCUSSION

We present a method of identifying, marking, and removing a radiolucent single-incision midurethral sling anchor placed into the obturator muscles. Among patients who undergo mesh removal of a single-incision midurethral sling, chronic pelvic pain is the indication in most patients. Unfortunately, pain persists in almost half of these patients.^3^ Groin pain can be a manifestation of obturator nerve entrapment, known to occur with transobturator sling procedures.^4^ In some people, the obturator nerve passes through the obturator internus before its division into the anterior and posterior branches.^5^ It is plausible that a single-incision midurethral sling anchor in the obturator internus muscle could irritate the obturator nerve. For this patient, because of the temporal development of pain shortly after placement of a single-incision midurethral sling, multiple treatment failures, and the plausibility of obturator nerve irritation, we advised removal of the retained anchor. Her pain was multifactorial, and she benefited from additional therapy for interstitial cystitis and pelvic floor muscle spasm.

We used MRI-guided methylene blue injection to localize the single-incision midurethral sling anchor. Similar staining techniques have been used to localize abnormal thyroid tissue with ultrasound, lung nodules with computed tomography, and intradural spinal lesions with biplanar fluoroscopy.^6–8^ Methylene blue staining has been done under ultrasound guidance to guide removal of radiolucent foreign bodies such as splinters and glass.^9^ Although inspired by previous image-guided marking techniques, this case is unique for its use of MRI-guided methylene blue localization. Although methylene blue is typically avoided in MRI because of its lack of signal visibility, this challenge was overcome by adding gadolinium to the mixture. Medicare reimbursements for this preprocedural MRI are $70.84 for physician billing and $345.36 for hospital billing. Private payer rates are variable but are substantially higher.

We decided on a transvaginal approach because the anchor was located primarily in the obturator internus muscle, 1.5 cm from the lateral border of the vagina. In contrast, an anterior groin approach would have involved a 6-cm-deep dissection carried through the hip adductors and the obturator membrane to reach the obturator internus muscle. The methylene blue staining in the obturator internus muscle next to the anchor was crucial to our success in removing it. Thoroughly embedded in muscular tissue, the anchor was palpable but not visible, and the blue marker allowed us to target a specific region. It is worth noting that the distance dissected from under the urethra to the plastic anchor was 5–6 cm, much deeper than the MRI-measured distance of 1.5 cm mentioned earlier, likely resulting from a change from prone to lithotomy positioning and bladder emptying after catheter insertion.

Two other case studies describe removal of single-incision midurethral sling anchors, one describing an anchor that eroded into the bladder requiring intravesical excision with endoscopic scissors.^10^ The other case study describes anchor removal in a patient who had a previous mesh removal.^11^ In this case, a spinal needle was inserted under guidance from an intraoperative curvilinear endocavitary ultrasound to target the anchor after dissection was completed up to the obturator internus. The dissection continued along the path of the needle until the anchor was reached. We used MRI because we thought that the small size and low density of the anchor would make it hard to see on ultrasound. In the future, we can image with ultrasound first with a low threshold for changing to MRI. This case demonstrates the importance of preoperative MRI for surgical planning and the feasibility of pairing MRI guidance with methylene blue dye marking to aid with visualization during surgery.
